# Deep learning-based scoring method of the three-chamber social behaviour test in a mouse model of alcohol intoxication. A comparative analysis of DeepLabCut, commercial automatic tracking and manual scoring

**DOI:** 10.1016/j.heliyon.2024.e36352

**Published:** 2024-08-28

**Authors:** Mohamed Aly Zahran, Aroa Manas-Ojeda, Mónica Navarro-Sánchez, Esther Castillo-Gómez, Francisco E. Olucha-Bordonau

**Affiliations:** a Unitat Predepartamental de Medicina, Facultat de Ciències de la Salut, Universitat Jaume I, Castellón de la Plana, Spain; b CIBERsam-ISCiii, Spain

**Keywords:** Sociability, Deeplabcut, Novelty, Artificial intelligence, 3-Chambers, Memory

## Abstract

**Background:**

Alcohol consumption and withdrawal alter social behaviour in humans in a sex-dependent manner. The three-chamber test is a widely used paradigm to assess rodents' social behaviour, including sociability and social novelty. Automatic tracking systems are commonly used to score time spent with conspecifics, despite failing to score direct interaction time with conspecifics rather than time in the nearby zone. Thereby, the automatically scored results are usually inaccurate and need manual corrections.

**New method:**

New advances in artificial intelligence (AI) have been used recently to analyze complex behaviours. DeepLabCat is a pose-estimation toolkit that allows the tracking of animal body parts. Thus, we used DeepLabCut, to introduce a scoring model of the three-chamber test to investigate alcohol withdrawal effects on social behaviour in mice considering sex and withdrawal periods. We have compared the results of two automatic pose estimation methods: automatic tracking (AnyMaze) and DeepLabCut considering the manual scoring method, the current gold standard.

**Results:**

We have found that the automatic tracking method (AnyMaze) has failed to detect the significance of social deficits in female mice during acute withdrawal. However, tracking the animal's nose using DeepLabCut showed a significant social deficit in agreement with manual scoring. Interestingly, this social deficit was shown only in females during acute and recovered by the protracted withdrawal. DLC and manually scored results showed a higher Spearman correlation coefficient and a lower bias in the Bland-Altman analysis.

**Conclusion:**

our approach helps improve the accuracy of scoring the three-chamber test while outperforming commercial automatic tracking systems.

## Highlights

• A deep learning procedure to analyze the 3-chamber test is introduced to extract data on sociability and social novelty.
• The method has been compared with manual scoring and commercial automatic tracking.
• Methods based on deep learning provide unbiased and reliable analysis of behaviour in the 3-chambers test.
• Analysis with DeepLabCut/SimBA correlates better to manual scores than commercial automatic tracking.

## 1 Introduction

The study of the effects of chemical compounds on behaviour depends to a great extent on having robust methods providing objective non-biased analysis of specific moments in behavioural performance of controlled environments and mazes. The three-chamber sociability and social novelty test is one of the most used paradigms to assess sociability preference in mice models [1]. In this paradigm, sociability preference is when the subject mouse spends more time interacting directly with the social target (mouse) than the inanimate novel object whereas social novelty preference is when the subject mouse spends more time interacting directly with the novel target (never met before) than the familiar target (met before). Acute alcohol administration promotes social preference in male and female mice [2,3] whereas withdrawal from alcohol reduces sociability preference in DBA male mice, but not in C57 male mice [4]. In the three-chambers studies, time scoring could be done manually by counting the duration of direct interactions (facing/sniffing) of the subject mouse with the stimulus target (empty, social, familiar, or novel target). Nevertheless, this method is very time-consuming and requires experienced annotators who can accidently introduce variability or bias [[5], [6], [7], [8], [9]]. Also, automated behaviour tracking software (e.g., Ethovision© by Noldus and Any-Maze© by Stoelting) could be used for time scoring, this kind of automatic tracking estimates the overall position of the whole animal body as a center of mass depending on the differences between the animal color and the background color [10] but not able to track single body parts (e.g. nose) [11] so that those automated tracking softwares are commonly used by most labs to score the spent time by the subject animal close to the stimulus target rather than the time of direct interaction [12]. In this context, automated scoring may give inaccurate results, for example, in case an animal stays or self-grooming close to the stimulus target without direct interaction, thereby, manual correction is still recommended to correct and confirm the automatically scored results [12]. New advances in artificial intelligence (AI) have made tracking an animal's different body parts possible. In this context, DeepLabCut (DLC) (https://github.com/DeepLabCut) [13,14], an open-source pose-estimation toolkit, allows tracking of multiple body parts of an animal. It trains a deep neural network to learn to anticipate poses using user-provided samples (extracted and annotated frames from provided videos) and generate tracking data (CSV/H5 files) of multiple body parts' positions, orientations, and movements. Thus, it would be possible to track a single body part like an animal's nose in the very close proximity to the stimulus target (1 cm around the cup) as a proxy for direct interaction (facing/sniffing) with the stimulus target. In this approach, Simple Behavioural Analysis (SimBA) [15] was used to analyze the tracking data generated by DeepLabCut.

Thus, this work aimed to introduce an analysis model of behaviour in the three-chambers test by using DeepLabCut/SimBA. We have checked this procedure to investigate alcohol withdrawal effects on sociability and social novelty preferences in mice considering sex and withdrawal periods (acute and protracted withdrawal). Additionally, we have compared the results of two automatic pose estimation methods: commercial automatic tracking (AnyMaze) and DeepLabCut considering the manual scoring method, the current gold standard.

## 2 Methods

### 2.1 Animals

Male and female mice (C57BL/6) at the age of 3–4 months and weight of 28–31 g were socially housed by sex after weaning (2–3 animals per cage) and then randomly distributed into four groups, Saline treated males (CTR-M), Ethanol treated males (ETH-M), Saline treated females (CTR-F), and ethanol-treated females (ETH-F). The stages of the estrus cycle were determined for the female mice using vaginal cytology and delegated to an experimental group to balance the saline and ethanol groups. Estrus cycle stages were identified by different cell types (nucleated epithelial, cornified epithelial, and leukocytes) that are linked to the different stages of the estrus cycle (proestrus, estrus, metestrus, and diestrus) [16]. The animals were kept under standard conditions (12:12 h light/dark cycle and ±21 °C; food and water). All animal procedures were approved by the Committee of Ethics and Animal Welfare of the Universitat Jaume I, Conselleria de Agricultura de la Generalitat Valenciana (procedure 2023-VSC-PEA-0227), and agreed with directive 86/609/EEC of the European Community on the protection of experimental animals.

### 2.2 Treatment

**Fig. 1:**
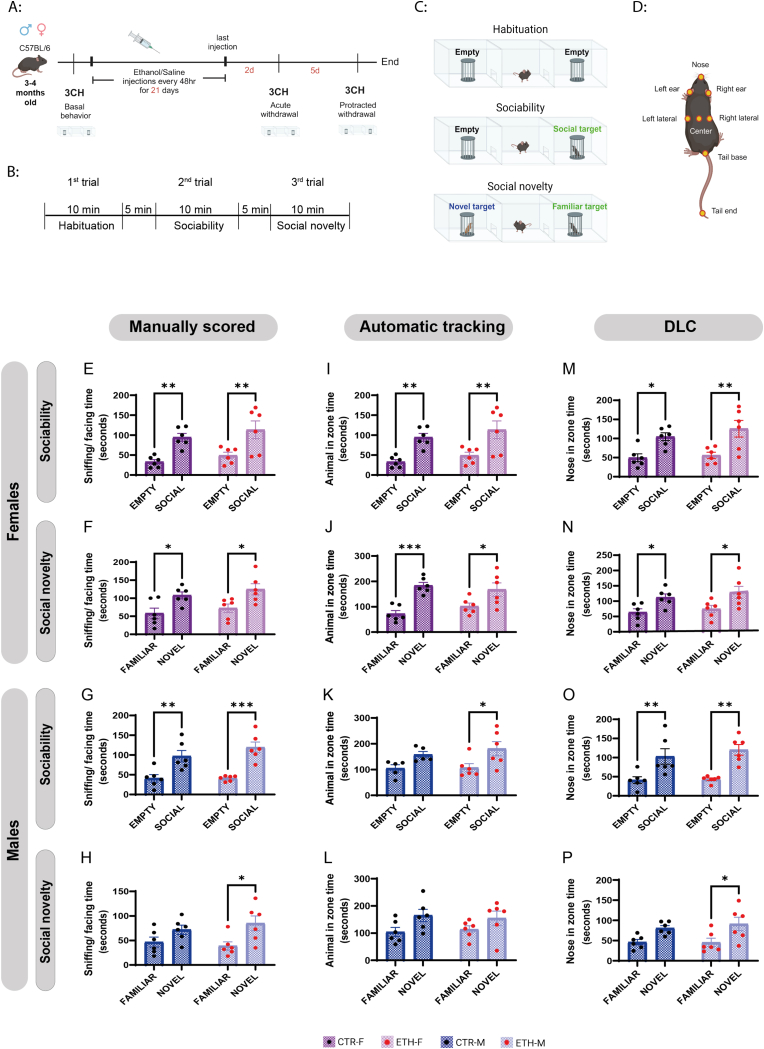
**Experimental design, three scoring methods showing preferences for the social target and the novel target in the three-chamber test at basal condition. A:** Experimental timeline. 3-4 months male and female mice were tested for sociability and social novelty preferences using the three-chamber test. They were then injected intraperitoneally either with diluted ethanol solution (2.4 g/kg) or the same volume of 0.9 % normal saline every other day for 21 days. Sociability and social novelty preferences were then tested during the acute and protracted withdrawal periods. **(B, C):** Illustration of the three-chamber test procedures including the habituation session in which both cups are empty, the sociability session in which a social target is represented in one cup, and the social novelty session in which a novel target is introduced with the familiar target. **D:** Illustrative figure of the mouse body parts annotations used for the DLC model. **(E, F, G, H):** Basal social target preference, and novel target preference evaluated by three-chamber test and manually scored. **(I, J, K, L):** Basal social target preference, and novel target preference scored by automatic tracking of the animal's whole body (AnyMaze). **(M, N, O, P):** Basal social target preference and novel target preference scored by DLC tracking of the animal's nose. In all graphs, each dot represents an animal, and data are expressed as the mean ± SEM (n = 6/group). Two-way ANOVA followed by Bonferroni post-hoc analysis; statistical significance: *p < 0.05, **p < 0.01, ***p < 0.001.

Ethanol, purchased from Panreac (Barcelona, Spain), was diluted to 20 % (v/v) with 0.9 % normal saline. Mice were injected intraperitoneally with either the diluted ethanol solution (2.4 g/kg) or the same volume of 0.9 % normal saline every other day for 21 days for a total of 10 injections. All injections were administered during daylight between 9 a.m. and 10 a.m. The weights of the animals were measured before treatment (ethanol or saline), 48 h after the last injection, and 7 days after the last injection (Fig. 1A).

### 2.3 Three-chamber sociability and social novelty test

The mice performed a three-chamber test to assess sociability and social novelty preferences. The test was performed before starting the treatment to represent the basal behaviour, 48 h after the last injections to represent the acute withdrawal behaviour, and 7 days after the last injection to represent the protracted withdrawal behaviour (Fig. 1A). All tests were performed between 10 a.m. and 1:00 p.m. and video recorded with a Logitech C615 HD webcam and OBS Studio v27.01 at 30 fps. The apparatus was an open clear acrylic box (19.5 cm × 40.2 cm x 60 cm) divided by two walls into three chambers (one central and two lateral). Each wall had a central door (7 × 7.2 cm) to form a channel between the three chambers. Each lateral chamber contained a wire cage on one side so that it was divided virtually into two zones of the same size in the video tracking: the empty zone (EZ) and the Holding Zone (HZ) where the wire cages were placed. The social, familiar, or novel animals were placed inside the perforated wire cages to allow sniffing but limit aggressive or sexual interaction between animals. The area around each wire cage was considered to analyze the time spent exploring the animal inside (WZ). The test occurred in three sessions of 10 min: (I) The first session was habituation in which the subject mouse adapted to the empty three chambers. (II) The second session was “sociability” in which the subject mouse encountered a social target (never-before-met) in a wired cage over one lateral chamber and an empty wired cage over the opposite lateral chamber. (III) The third session was “social novelty” in which the wire cage enclosing the familiar mouse (the social target in the sociability session) was moved to the opposite lateral chamber that had been empty during the sociability session or was kept in his chamber, and a second novel mouse (never-before-met) was placed in the other lateral chamber in a wired cage. The subject mouse was free to explore either the social target or the empty cage in the sociability session, and the familiar mouse or the novel mouse (unfamiliar) in the social novelty session (Fig. 1B–C). After, each session the subject mouse was removed from the apparatus and gently returned to its home cage inside the room of the experiment for 5-min break and during that time the apparatus and the cups were wiped down with 70 % ethanol to remove any residual odours that may affect subsequent session/test. The time the subject mouse spent in each chamber and the time spent sniffing each wire cage were measured in each session. The sociability preference index was calculated from the following formula [(time exploring the social target-time exploring the empty target)/(time exploring the social target + time exploring the empty target)] whereas the social novelty preference index was [(time exploring the novel target-time exploring the familiar target)/(time exploring the novel target + time exploring the familiar target)].

### 2.4 Video analysis

Video analysis was performed manually by an experimentally blind researcher, and the time spent by the subject animal sniffing each cup was measured. Additionally, videos were analyzed using both AnyMaze (Stoelting, Wood Dale, IL, USA) and DeepLabCut/SimBA deep learning algorithms.

#### 2.4.1 Video analysis with anymaze

Anymaze behaviour tracking software (version 7.4) (Stoelting, Wood Dale, IL) was used. As the software tracks the animal as a center of mass, a wide ROI was drawn around each wired cup (WZ, about 5 cm around the cup) (Fig. 3E) to be suitable for tracking the subject animal's body in close proximity to each wired cup. The amount of time spent by the subject animal inside each ROI (close to the cup rather than direct interaction)was measured. Additionally, Anymaze software enables tracking of the animal's head, it uses the center of masses as the key reference point to plot the head or the tail positions, so that we tracked the animal's head and scored the spent time in close proximity to each wired cup (about 1 cm around the cup) as a proxy for interaction time.

#### 2.4.2 Video analysis using DeepLabCut/SimBA

DeepLabCut (version 2.3.8) [13,14] was used for body parts tracking. A step-by-step protocol for installation and work procedures is available on their website (https://github.com/DeepLabCut). For DeepLabCut/SimBA analysis we have used the following protocol: (1) Labeling of animal body parts; a total number of 200 frames from 10 videos were extracted using k-means algorithm and labeled with eight key points of the body (left ear, nose, right ear, center, left lateral, right lateral, tail base & tail end) (Fig. 1D). (2) Network training and validation; the network was trained and evaluated using Google Colaboratory Notebook; the training fraction was set to 0.95, and the batch size was set to 8. The network was trained for 140 K iterations using ResNet_50 pre-trained network and validated with 1 number of shuffles, the train and test errors were 2.44 and 3.31 pixels, respectively, and 2.44 and 3.28 pixels respectively with P cutoff of 0.6 (3) Video analysis and defining region of interest (ROI); the validated network was used to analyze all videos of the three-chamber test. Labeled videos were created, and visual inspection confirmed accurate tracking of all body parts. Simple Behaviour Analysis (SimBA) toolkit version 1.87.2 (https://github.com/sgoldenlab/simba) [15] was used to analyze DeepLabCut tracking data; a very narrow ROI was drawn around each wired cup (about 1 cm around the cup) to be suitable for tracking the subject animal's nose but not the whole body. (4) Extracting data; The amount of time spent by the subject animal's nose inside the ROI (facing/sniffing each wired cup) was measured. The trained model with instruction to reuse is available at https://osf.io/b8g7w/.

#### 2.4.3 Statistics

Statistical analysis was performed using the GraphPad Prism 9 software (GraphPad Software Inc., La Jolla, CA, USA). Normality of the data was assessed using the Shapiro-Wilk test. Two-way analysis of variance (ANOVA) followed by post-hoc Bonferroni's multiple comparison test was used to compare the different experimental groups. Spearman correlation was performed by using a confidence interval of 95 % and measuring linear regression to correlate automatically (AnyMaze) scored and DLC-scored results with manually scored results. A Bland-Altman Plot analysis was performed to evaluate the agreement levels between the two scoring methods and manual scoring. The α value was set at 0.05 for all analyses. Statistical significance was established at p < 0.05 (*p < 0.05, **p < 0.01, ***p < 0.001, ****p < 0.0001). Supplementary tables contain details of the statistical analysis.

## 3 Results

We have made the analysis on 6 animals per group for each experimental condition. The Shapiro-Wilk analysis of normality showed that all groups passed the normality test. Thus, a comparative ANOVA can be applied.

### 3.1 All animals preferred the social target and novel target in the basal condition

Mice are social species that have a natural tendency to approach social stimulus and investigate novel (unfamiliar) conspecifics [17]. Therefore, all animal groups (CTR-F, ETH-F, CTR-M, & ETH-M) were tested for sociability and social novelty preferences using the three-chamber test before starting the alcohol intoxication protocol to confirm normal behaviour among the experimental groups. The results are referred to as basal behaviour. Both male and female mice showed a significant preference for the social target during the sociability session, and for the novel target during the social novelty session. Two-way ANOVA revealed a significant target effect (females sociability: P = 0,0001, F (1, 20) = 22,85; females social novelty: P = 0,0009, F (1, 20) = 15,32; males sociability: P < 0,0001, F (1, 20) = 37,22; males social novelty: P = 0,0044, F (1, 20) = 10,28) and no group effect (CTR vs ETH) (Supplementary Table 1). Bonferroni's post hoc analysis showed a significant preference for the social target during the sociability session (Fig. 1E,G) and a preference for the novel target over the familiar target during the social novelty session in the basal condition (Fig. 1F and H). There were no differences in sociability or social novelty levels between the control and ethanol groups under basal conditions in either male or female mice (Supplementary Table 1) (Fig. 1E:1H, 2A: 2D).

### 3.2 Sex-specific effect of alcohol withdrawal on sociability and social novelty preferences

**Fig. 2:**
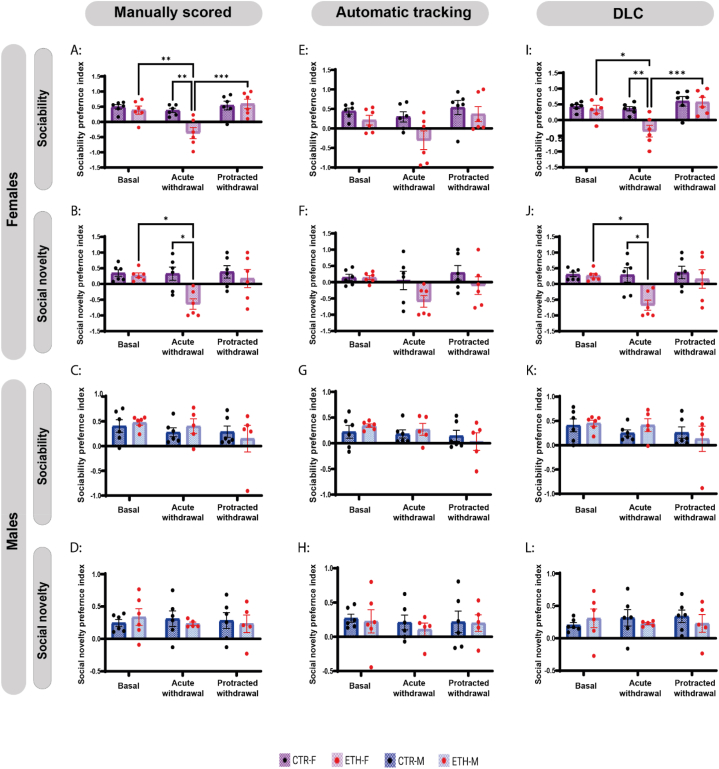
**Only female mice showed sociability and social novelty deficits during acute alcohol withdrawal. The AmyMaze scoring method failed to represent the significance of these deficits; however, the DLC scoring method did. (A, B)** Alcohol-treated females showed significant deficits in sociability and social novelty compared with the control group during acute withdrawal. At the protracted withdrawal, the sociability deficit was significantly restored to the basal level whereas the social novelty deficit was insignificantly restored. **(C, D):** Alcohol-treated males didn't show any difference in sociability or social novelty preferences during the acute and protracted withdrawal. **(E, F, G, H):** Sociability and social novelty preferences at basal, acute withdrawal, and protracted withdrawal scored by automatic tracking of the animal's whole body (AnyMaze) failed to show any significance in the data. **(I, J, K, L):** Sociability and social novelty preference at basal, acute withdrawal, and protracted withdrawal scored by DLC tracking of the animal's nose showing females' sociability and social novelty deficits during acute withdrawal in agreement with manually scored data. In all graphs, each dot represents an animal, and data are expressed as the discrimination index mean ± SEM (n = 5–6/group). Two-way ANOVA followed by Bonferroni post-hoc analysis; statistical significance: *p < 0.05, **p < 0.01, ***p < 0.001.

Sociability and social novelty were assessed during acute and protracted withdrawal periods. Two-way-ANOVA has revealed significant period and group effects on female mice sociability (P = 0,0005, F (2, 30) = 9807; P = 0,0202, F (1, 30) = 6015; respectively) and social novelty session (P = 0,0317, F (2, 30) = 3883; P = 0,0112, F (1, 30) = 7,298, respectively). Using Bonferroni's post hoc analysis, alcohol group showed a significant deficit in sociability (P = 0.006) and social novelty (P = 0.015) vs control group during the acute withdrawal (Fig. 2A and B), and by the protracted withdrawal, this sociability deficit was significantly restored to the basal level (P = 0.0003) (Fig. 2A) whereas the social novelty deficit was insignificantly restored (P = 0.069) (Fig. 2B) (Supplementary Table 1). Interestingly, no such alterations in sociability and social novelty behaviours were observed in male mice during the different withdrawal periods (Fig. 2C and D).

### 3.3 The DeepLabCut results outweigh the commercial automatic tracking results (AnyMaze)

**Fig. 3:**
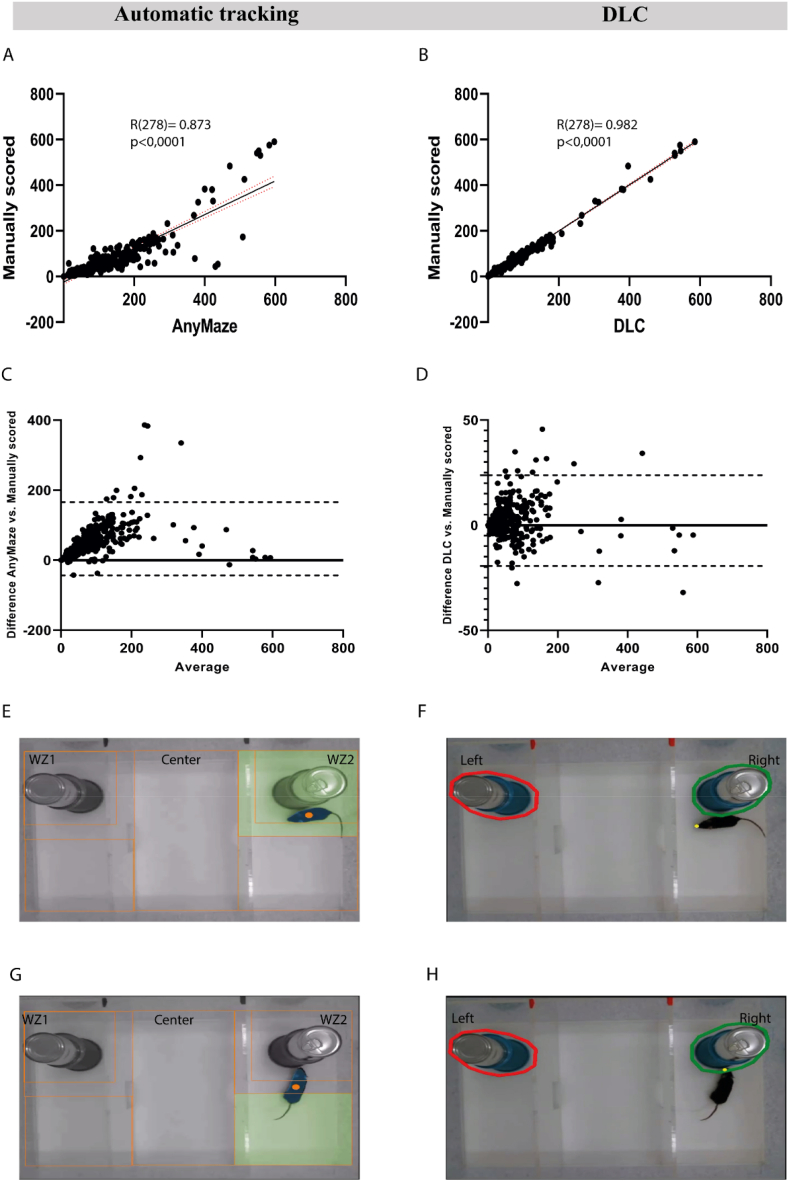
**Both the automatic tracking and DLC-scored results were in agreement with the manually scored results. However, the DLC method outperforms the AnyMaze method. (A, B):** Spearman correlation of automatic tracking results (A) and DLC (B) results vs manually scored results. The correlation coefficients and significance are shown on the graph. **C:** Bland-Altman plot of automatic tracking results and manually scored results showing bias values of 61.31, Sd = 53.40, and 95 % limits of Agreement of −43,35 (lower dashed line) and 166,0 (upper dashed line). **D:** Bland-Altman-Plot of DLC results and manually scored results showing bias value of 2.23, Sd = 11.02, and 95 % limits of Agreement of −19,36 (lower dashed line) and 23,83 (upper dashed line). **(E, G):** Screenshots of an automatic tracking method (AnyMaze) tracking animal body as a centre of mass and showing false positive (E) and false negative (G) scoring of interaction with the wired cup. The WZ1 and WZ2 zones were 5 cm around the wired cup to be suitable for tracking the entire body of the animal in close proximity to the wired cup. The orange dot represents the center position of the animal's body detected by AnyMaze. **(F, H):** Screenshot of the DLC tracking method (one body part “nose”) showing negative (F) and positive (H) scoring of direct interaction with the wired cup. The left and right zones were 1 cm around the wired cup to track the animal's nose. The yellow dot represents the animal's nose detected by DLC. (For interpretation of the references to color in this figure legend, the reader is referred to the Web version of this article.)

The tracking precision of DeepLabCut has already been demonstrated by its developers [13] so we used DLC tracking of a single body part (nose) along with a commercial automatic tracking system of the animal's whole body (AnyMaze) as a proxy for animals' interactions in the three-chamber test. The results of the two different scoring methods, DLC and automatic tracking, were cross-referenced and compared to the manually scored results. Both scoring methods showed preferences for the social target and the novel target at the basal behaviour of all animals, in agreement with the manually scored results; however, the DLC results showed more similarity to manually scored results. (Supplementary Table 3) (Fig. 1I:1L, 1M:1P). The DLC scoring method showed significant deficits in sociability and social novelty, as indicated by the manually scored results in female mice during acute withdrawal (Fig. 2I and J) whereas the automatic tracking scoring method showed insignificant deficits (Fig. 2E and F). Spearman correlation analysis revealed that the results from both scoring methods, DLC and automatic tracking, showed significantly high correlations with the manually scored results (R(278) = 0.982, P < 0,0001; R(278) = 0.873, P < 0,0001; respectively), with a higher correlation coefficient of the DLC results (Supplementary Table 5) (Fig. 3A 3B). Additionally, the descriptive analysis of Bland-Altman revealed that the results from both scoring methods were in good agreement with the manually scored results. However, DLC and manually scored results had lower bias values (bias = 2.23, Sd = 11.02, 95 % agreement limits: 19,36:23,83) compared to automatic tracking and manually scored results (bias = 61.31 S d = 53.40, 95 % agreement limits: 43,35:166,0) (Supplementary Table 5) (Fig. 3C and D).

## 4 Discussion

Several studies have shown that social deficits, which are related to an inability to maintain amicable relationships are the main features of many psychiatric disorders such as autism spectrum disorder [18], social anxiety [19,20] schizophrenia [21] and addiction [22]. Therefore, in mice models of those disorders, measuring social deficit is crucial for assessing pathological phenotypes and developing treatment strategies [23]. In this approach, the three-chamber test is widely used to evaluate social behaviours in mice including sociability and social novelty preferences [12], especially in ASD research [17,[24], [25], [26], [27], [28], [29]]. Since the three-chamber test was developed in 2004 [1,30,31], many automatic scoring methods have been introduced. One method involves the use of photocells embedded in each doorway to track the time spent in each chamber [1,17]. Another possibility is the use of automated tracking software (for example AnyMaze and Ethovision), which estimates the overall position and tracks the time spent by the whole animal body in close proximity to the wired cup, rather than the entire chamber [12]. However, neither method was able to detect direct interaction (sniffing/facing) of the subject mouse with the wired cup Luxem et al., 2023; [10], chamber time/animal in zone time, and direct interaction time usually correlates positively with each other. Nevertheless, there are many occasions when the chamber time/animal in zone time is insignificant, but the direct interaction time is significant. Therefore, manual scoring is recommended to correct false positive and false negative scoring, and verify the automatically scored results, such as when the subject animal remains in the vicinity of the wired cup without direct interaction (Fig. 3E) or software tracking errors (Fig. 3G) [12,17]. Alternaively, tracking animal's head inside the ROI could be a proxy for the interaction time, AnyMaze enables tracking of the animal's head by using the center of mass as the key reference point to plot the head or the tail positions [10], this kind of body parts tracking usually results in frequent mismatching and head/tail swapping (Supplementary Figs. 1K–L) which resulted in less accurate results and lower Spearman correlation coefficient with the manually scored results compared to chamber time/animal in zone time method (Supplementary Tables 4–5) (Supplementary Fig. 1I). In this approach, DeepLabCut, a pose-estimation toolkit that allows the tracking of an animal's multiple body parts [13], enabled the tracking of the subject animal's nose in very close proximity to the wired cup as a proxy for the time spent in interaction rather than chamber time/animal in zone time tracking (Fig. 3F and H, see also supplementary video 1 and 2). While it is likely that an interaction (facing/sniffing) is occurring if the subject animal's nose is in the tight ROI around the cup, this method cannot exclude moments when the animal's nose is in the ROI, but no interaction is taking place at that time. This limitation applies to both automated behaviour tracking software and DeepLabCut.

Supplementary video related to this article can be found at https://doi.org/10.1016/j.heliyon.2024.e36352

The following is/are the supplementary data related to this article:

Video 1 2 Video 1

Video 2 Video 2

In this study, we have found that commercial automatic tracking systems for an animal's whole body/head (eg. AnyMaze) has failed to detect the significance of the social deficits shown by manual scoring in female mice during acute withdrawal from alcohol. However, tracking the animal's nose using DeepLabCut showed a significant social deficit, in agreement with manual scoring. Interestingly, this social deficit was shown exclusively in female mice during acute withdrawal from alcohol and was recovered by the protracted withdrawal suggesting a starting point for investigating the underlying neural mechanism of this sex-dependent impairment and introducing treatment targets. Additionally, DLC and manually scored results have shown a higher Spearman correlation coefficient and a lower bias in the Bland-Altman analysis which confirms that using DLC in tracking the animal's nose, outweighs the commercial automatic method of animal whole-body/head tracking in scoring the three-chamber sociability and social novelty test. Moreover, DLC has a fast-growing community that enables data sharing and reuse among laboratories to train and improve networks. In addition, the tracking data on the position and movement of the animal's multiple parts could be further analyzed using a supervised machine-learning approach to quantify complex behaviours such as self-grooming, rearing, or jumping. One of the most widely used toolkits for this purpose is SimBA [15]. Another approach to further analysis of DLC tracking data could be an unsupervised machine-learning approach to discover and cluster new animal behaviour patterns, such as B-SOiD [[32], [33]].

## Data availability statement

Raw data that support the findings of this study are deposited at https://zenodo.org/records/11453185 DOI 10.5281/zenodo.11453185″ title = "doi:DOI 10.5281/zenodo.11453185">DOI 10.5281/zenodo.11453185.

## CRediT authorship contribution statement

**Mohamed Aly Zahran:** Writing – review & editing, Writing – original draft, Software, Methodology, Investigation, Formal analysis, Data curation, Conceptualization. **Aroa Manas-Ojeda:** Writing – review & editing, Software, Methodology, Formal analysis. **Mónica Navarro-Sánchez:** Writing – review & editing, Software, Methodology, Conceptualization. **Esther Castillo-Gómez:** Writing – review & editing, Supervision, Project administration, Funding acquisition, Conceptualization. **Francisco E. Olucha-Bordonau:** Writing – review & editing, Writing – original draft, Supervision, Resources, Project administration, Methodology, Funding acquisition, Formal analysis.

## Declaration of competing interest

The authors declare no competing interests in this paper.
