# Supplementary material for: Deep learning-based scoring method of the three-chamber social behaviour test in a mouse model of alcohol intoxication. A comparative analysis of DeepLabCut, commercial automatic tracking and manual scoring
Source: Heliyon. 2024 Aug 28;10(17):e36352. doi: 10.1016/j.heliyon.2024.e36352 (PMC11403434; doi:10.1016/j.heliyon.2024.e36352)
Supplement: Multimedia component 1 [file mmc1.docx]

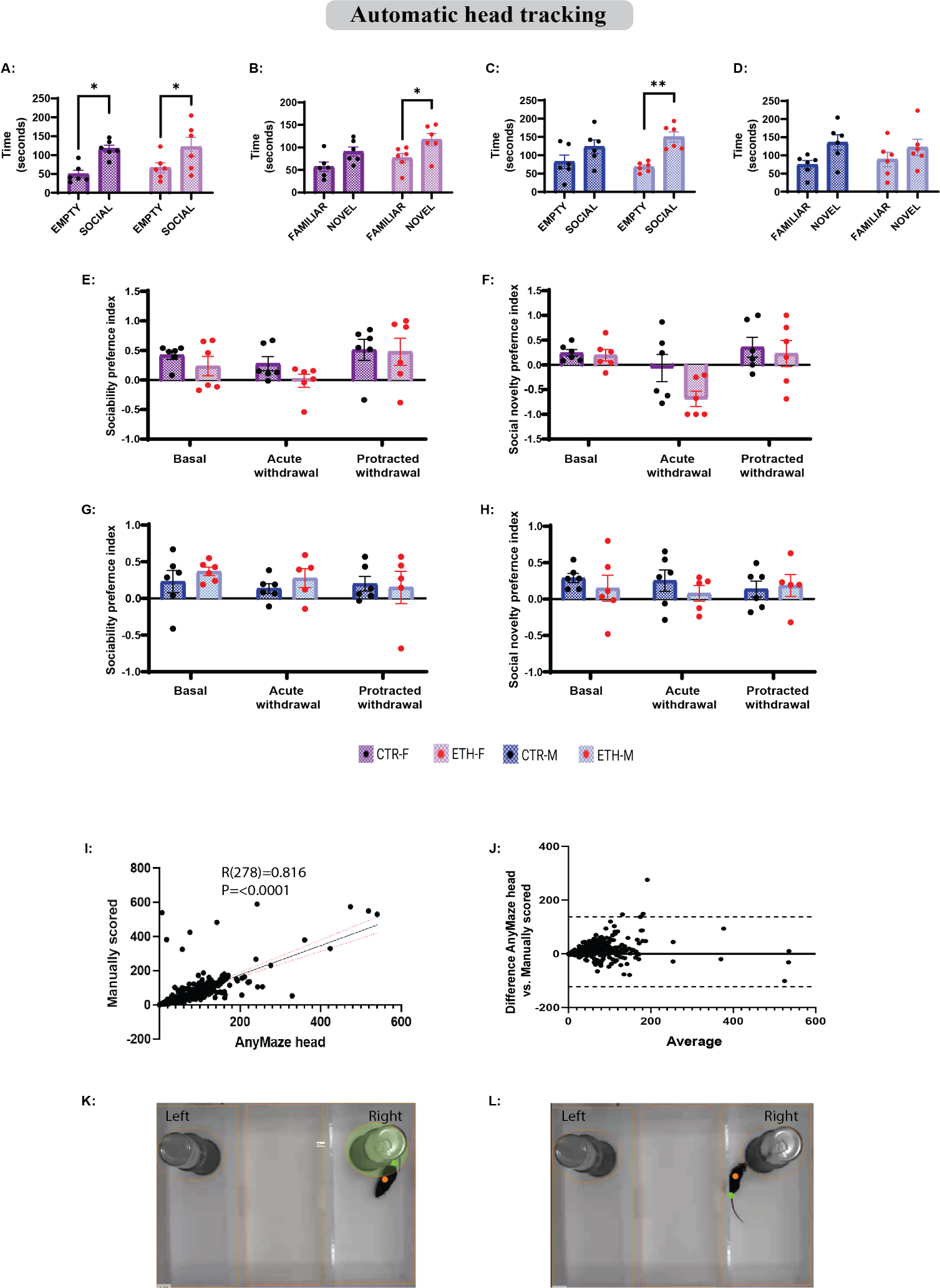


**Supplementary Figure 1: Automatic head tracking by AnyMaze showed frequent head/tail swapping which resulted in less accurate results and lower correlation with the manually scored results. (A, B, C, D):** Basal social target preference, and novel target preference scored by automatic tracking of the animal’s head (AnyMaze). **(E, F, G, H):** Sociability and social novelty preferences at basal, acute withdrawal, and protracted withdrawal scored by automatic tracking of the animal’s head (AnyMaze) failed to show any significance in the data. **I:** Spearman correlation of automatic head tracking results vs manually scored results. The correlation coefficients and significance are shown on the graph. **J:** Bland-Altman plot of automatic head tracking results and manually scored results showing bias values of 7.78 , Sd=66.50, and 95% limits of Agreement of -122.6 (lower dashed line) and 138,0 (upper dashed line). **(K, L):** Screenshots of an automatic tracking method (AnyMaze) tracking animal´s head showing head/tail swapping results in false positive (K) and false negative (L) scoring of interaction with the wired cup. The left and right zones were 1 cm around the wired cup to track the animal’s head. The orange dot represents the center position of the animal´s body while the green dot represents the automatic detection of the animal´s head by AnyMaze. In graphs (A-H), each dot represents an animal, and data are expressed as the mean ± SEM (n=5-6/group). Two-way ANOVA followed by Bonferroni post-hoc analysis; statistical significance: *p<0.05, **p<0.01, ***p<0.001.

Supplementary Table. 1

Supplementary Table. 2

Supplementary Table. 3

Supplementary Table. 4

Supplementary Table. 5
